# Using climatic variables alone overestimate climate change impacts on predicting distribution of an endemic species

**DOI:** 10.1371/journal.pone.0256918

**Published:** 2021-09-02

**Authors:** Somayeh Zangiabadi, Hassan Zaremaivan, LIuis Brotons, Hossein Mostafavi, Hojjatollah Ranjbar

**Affiliations:** 1 Department of Plant Biology, Faculty of Biological Sciences, Tarbiat Modares University, Tehran, Iran; 2 CREAF, Cerdanyola del Vallès, Spain; 3 InForest Jru (CTFC-CREAF), Solsona, Spain; 4 CSIC, Cerdanyola del Vallès, Spain; 5 Department of Biodiversity and Ecosystem Management, Environmental Sciences Research Institute, Shahid Beheshti University, Tehran, Iran; 6 Department of Mining Engineering, Shahid Bahonar University of Kerman, Kerman, Iran; Qinghai University, CHINA

## Abstract

Plant species distribution is constrained by both dynamic and static environmental variables. However, relative contribution of dynamic and static variables in determining species distributions is not clear and has far reaching implications for range change dynamics in a changing world. *Prunus eburnea* (Spach) Aitch. & Hemsl. is an endemic and medicinal plant species of Iran. It has rendered itself as ecologically important for its functions and services and is currently in need of habitat conservation measures requiring investigation of future potential distribution range. We conducted sampling of 500 points that cover most of Iran plateau and recorded the *P*. *eburnea* presence and absence during the period 2015–2017. In this study, we evaluated impacts of using only climatic variables versus combined with topographic and edaphic variables on accuracy criteria and predictive ability of current and future habitat suitability of this species under climate change (CCSM4, RCP 2.6 in 2070) by generalized linear model and generalized boosted model. Models’ performances were evaluated using area under the curve, sensitivity, specificity and the true skill statistic. Then, we evaluated here, driving environmental variables determining the distribution of *P*. *eburnea* by using principal component analysis and partitioning methods. Our results indicated that prediction with high accuracy of the spatial distribution of *P*. *eburnea* requires both climate information, as dynamic primary factors, but also detailed information on soil and topography variables, as static factors. The results emphasized that environmental variable grouping influenced the modelling prediction ability strongly and the use of only climate variables would exaggerate the predicted distribution range under climate change. Results supported using both dynamic and static variables improved accuracy of the modeling and provided more realistic prediction of species distribution under influence of climate change.

## Introduction

Anthropogenic climate change will affect biodiversity in different parts of world rapidly [1] and lead to large or small reductions or enhancements in species distribution areas [2]. Since, species cannot adapt as fast as environmental changes taking place, they change their distribution range and shift to new habitats or go extinct [3, 4]. Investigation on the effects of climate change on habitat suitability of species contributes greatly to the conservation of biodiversity [5]. A key issue in management of environmental resources and conservation of biodiversity is comprehension of how and how much future climate affects the species occurrences [6] especially, on endemic and endangered species [7].

Species distribution modelling (SDM) is a prominent method for predicting effects of climate change on potential distribution of species based on correlations between presence or presence and absence information of species and informative environmental predictors as driving predictors [8]. Habitat suitability maps are the output of species distribution models [9] that have been used widely for different purposes, such as predicting niche shifts of nonnative plants [10], assessment of climate change impacts on species distribution [4], testing ecological, biogeographical and evolutionary hypotheses [11, 12], predicting distributional changes at expanding range margins [13], planning future conservation [5], and also in assessing the statues of endemic and rare species under different scenarios of climate change [14]. Rare and endemic species with specialized habitat requirements represent a particular challenge for statistical analysis and valuable opportunity for predictive modelling plant species distribution [15].

Nowadays, a large number of environmental datasets are produced for different studies, such as SDM which most of them are statistically dependent [16]. Using appropriate number of variables has always been a goal to avoid prediction errors. For example, use of a large number of variables in SDM may led to collinearity problems between variables [17] and overfitting [18, 19] and deficient use led to overestimation in predictions [20]; therefore, in both cases their results are far from reality and mostly lead to under- or over-estimation in predictions [20].

One of the main reasons for credibility of modelling results is entailing (including) appropriate environmental variables into the model [9]. This is a direct consequence of the outputs of SDM are completely dependent on chosen input variables [4]. Therefore, using the most efficient and biologically relevant variables for modelling species distribution is a crucial step because it affects model performance, accuracy and reality of predictions [20, 21–26].

The species niche is affected by a number of biotic and abiotic factors at local and regional scale. However, biotic factors are often ignored in SDM because of the difficulty of task of meticulous data collection and quantification, leading to SDM most typically focusing on abiotic data instead. The main assumption of SDM is that effect of climate (dynamic, short-term, immediate) in species occurrence and distribution is a surrogate for other environmental (static, long-term) variables and therefore; climate directly or indirectly is a major constrain of species distribution [27]. For this reason and since collecting climate data is readily accessible and easily applicable in model processing [28], a common approach in many distribution modelling investigations has been to use only climatic variables [29, 30] with disregard to other environmental variables [20]. But, are these predictions solely based on climate data reliable and able to describe a realistic state of the environment capable of capturing species environmental constraints?

Environmental variables, such as soil properties and topographic profiles change only at long time scales [31] and as such, are considered as static in most SDM applications. Conversely, climatic variables are considered as dynamic variables because they change over shorter time periods. In the light of high speed of climate change in recent years and increase in frequency and intensity of extreme events (fires, floods and landslides), these static variables (soil and topography) can become non-static in some areas [6, 31]. Knowledge on plant species distribution and its relationship with dynamic and static environmental variables is growing [20]. However, determining relative contribution of each of dynamic (climate) and static (soil and topography) variables in predicting species distribution range under changing circumstances, such as prolonged climate change needs further research.

Here, we conducted the investigation with the following objectives: (1) to assess the impacts of using static (topographic and edaphic) variables combined with climatic (dynamic) variables on the accuracy of predictive potential distribution of a restricted distribution species *Prunus eburnea* (Spach) Aitch. & Hemsl. (gray almond) using species distribution models; and (2) to evaluate the effects of using different groups of environmental variables (static vs dynamic) on the spatial prediction of future distribution of *P*. *eburnea* under climate change; (3) to identify the main environmental variables that define variations in *P*. *eburnea* habitats, and (4) to assess the independent and joint effects of each environmental variable in the potential *P*. *eburnea* habitats. We expect that the use of climate only variables will render different future distribution patterns from the species when compared to models in which both dynamic but also static key environmental variables are included.

## Materials and methods

### Study area and species

**Fig 1:**
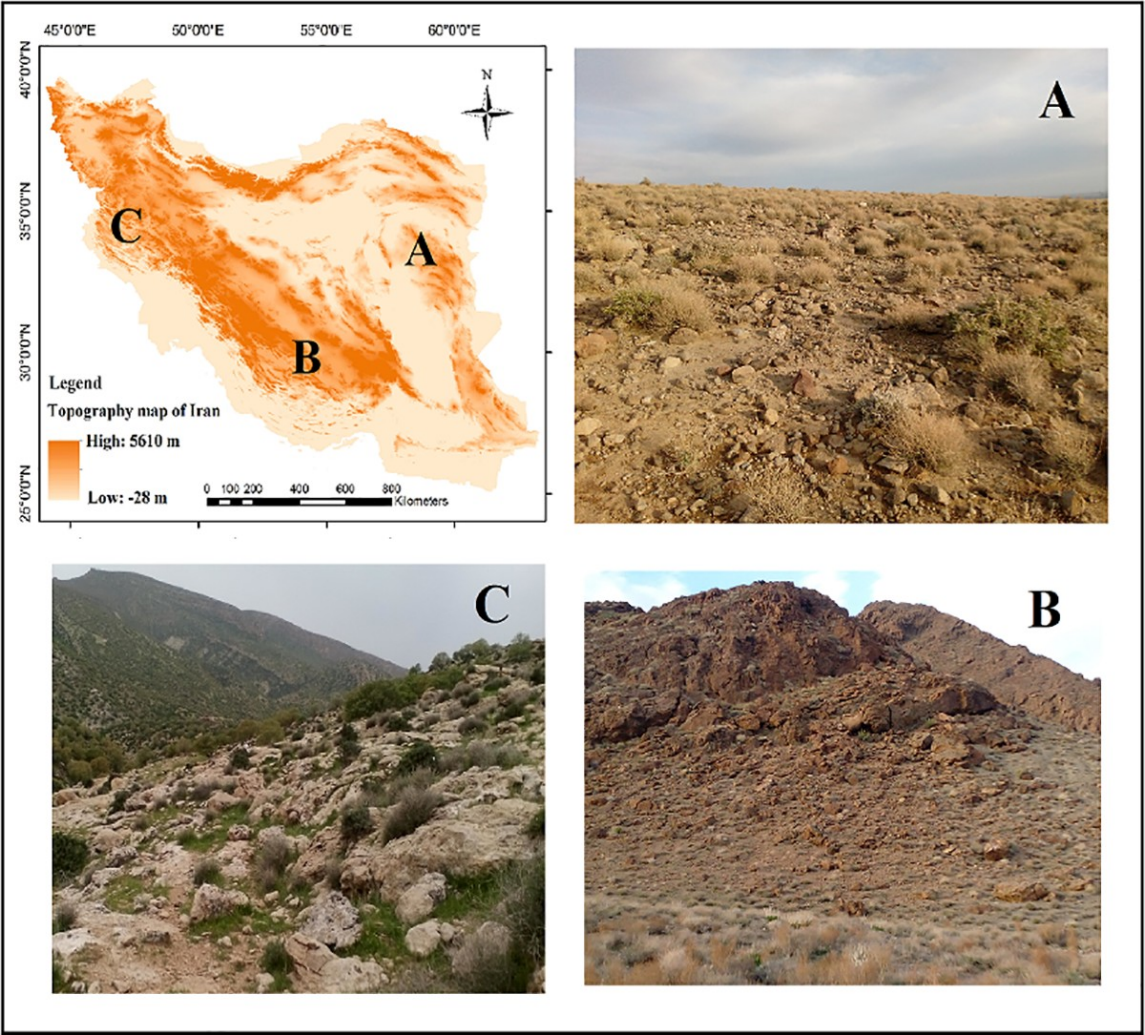
Location and landscape of three habitats of gray almond in east (A), south (B) and west (C) of Iran on topographic map.

*Prunus eburnea* (Spach) Aitch. & Hemsl. (gray almond) is a member of the subgenus Dodecandra in the genus *Prunus*, tribe Prunoideae from Rosaceae family [32]. It is an endemic and medicinal species, widely distributed in Irano-Touranian phytogeographical region of Iran [33]. Gray almond is distributed along the eastern, southern and southwestern borders of Iran, primarily grows in rocky, sandy limestone on high mountains and lower plateaus (Fig 1). Gray almond provided important services in soil erosion prevention and deforestation [34]. However, knowledge on the occurrence and distribution of this species is limited to the locations of collection by field researchers and little is known about its distribution and niche characteristics.

### Sampling

Main habitat requirements and range for *P*. *eburnea* were obtained from Flora of Iran [32], Flora Iranica [35] and Flora of Iran [36] studying specimens at herbariums, personal collections and scientific literature. From these sources, and the geographic information collected therein, we identified 52 areas in Iran in which the presence of *P*. *eburnea* was likely. In each of the areas, of roughly comparable size for which *P*. *eburnea* was potentially present, we placed from 4 to 5 random sampling stations, which were visited during 2015–2017. In areas outside potential species habitat, we conducted specific visits using an additional set of sampling stations in which the absence of the species was confirmed after visiting the area [37]. The minimum distance between sample stations was 10 kilometers. All sampling stations were far (10 km) from the cities and roads in order to reduce biases derived from the exploitation of the species around human settlements. Finally, a total of 500 sampling points of presence (230 N) and absence (270 N) of this species were recorded by using a portable Garmin S76, GPS model in the study area

### Environmental variables

We used available climatic and topographic data obtained from www.worldclim.org [38] and edaphic layers from SOILGRIDS.ORG [39] for describing the environmental space used to identify *P*. *eburnea* habitats. All environmental variables were in latitude/longitude projection and 1×1 km grid cells, which represented the minimum planning units of analysis for environmental layers for the study area. These were preprocessed, standardized and adjusted in the "Raster" library [40]. To avoid overfitting and collinearity problems between descriptor variables [41, 42], Pearson correlation test [43] was applied and finally 12 variables from different groups of environmental variables were retained (Table 1). Topographic layers were generated by using arcgeomorphometry tools [44] in ArcGIS 10.1 software [45].

**Table 1:** Layers of environmental variables used in this study.

Variable	Description
Bioclimat variables (19) *	Bio 3 = Isothermality (Bio 2/ Bio 7) (* 100)
(www.worldclim.org)	Bio 4 = Temperature seasonality (standard deviation *100) (°C)
	Bio 11 = Mean temperature of coldest quarter (°C)
	Bio 15 = Precipitation seasonality (coefficient of variation) (mm)
	Bio 17 = Precipitation of driest quarter (mm)
Topographic variables (3)	Topographic variables (slope, slope aspect, elevation)
(www.worldclim.org)	(Derived from DEM using Arc GIS 10.1 software)
Mean solar radiation (12)	Solar radiation (kJ m^-2^ day^-1^) per month
(www.worldclim.org)	
Soil	Coarse fragments (volumetric) (cm3 cm−3)
(https://soilgrids.org)	Organic carbon content (g kg-1)
	Silt content (gravimetric) (kg kg-1)

### Statistical analyses

Environmental layers of ecological importance for this species were selected according to available ecological knowledge about the species and expert opinions then linear correlation coefficient was done [46]. Layers with correlation less than 0.7 were selected. Then, variables that did not contribute significantly (p<0.005) to the explained variation were omitted [47]. PCA was used for exploring environmental patterns of data and discovering their relationship [46] as PCA considers the Euclidean distance among points when responses of species and environmental variables have linear relationship with each other [48]. We used PCA and the latent root criterion to reduce the number of variables and having uncorrelated components that accounted for most of the total variance among sampling points [49]. PCA analysis was performed by using ‘pcaMethods’ library [50] in R software [51] and twelve environmental variables finalized as shown in Table 1.

### Partitioning methods

Next, we aimed at assessing how much of the variation in habitat selection was explained by different groups of environmental variables. Decomposition and assembling of variables was carried out for better understanding of the relative importance of each environmental variable, group of variables and their joint effects [47]. Then, we performed hierarchical partitioning (HP) and Variation partitioning (VP) analyses. For evaluating the impacts of different aspects of ecosystems on presence of species, hierarchical partitioning approach was an appropriate method here as it provided a comprehensive and flexible framework for analyzing ecological questions at different levels of studies and across temporal (years) and spatial (geography) scales [52].

We performed a series of (partial) regression analyses with redundancy analysis (RDA) and hierarchical partitioning using the ‘hier.part’ library [53] and ‘gtools’ library [54] in R software [51]. Statistical testing for each added environmental variable was performed with the Monte Carlo permutation tests (9999 permutations). Hierarchical partitioning basis on monotonic relationships between the response variable (presences and absences) and variables [47] was done. For determining the importance of each group of environmental variables, we divided all environmental variables in terms of: (1) edaphic, (2) topographic and (3) climatic groups that are not shared by the other environmental groups. This would help to understand the ecological patterns in terms of independent and joint contribution of each variable, because it quantifies variations among different aspects of environmental components more precisely [46].

Variation contribution of individual variables in species distribution can be tested and determined by using Variation partitioning (VP) [55]. VP method was done and venn diagram was constructed by using ‘vegan’ library [56] in R software [51]. Adjusted R2 was measured which indicates the proportion of total variation [57]. In this study, VP led to eight fractions (I) pure effect of topographic variables; (II) pure effect of climatic variables; (III) pure effect of edaphic variables; and combined variation due to the joint effects of (IV) climatic and edaphic variables; (V) climatic and topographic; (VI) topographic and edaphic; (VII) the three groups of explanatory variables and finally (VIII) unexplained variation.

Variation partitioning and decomposition of variables can be visualized by a venn diagram. By decomposition of variables, we were able to compare the extent of variables redundancy and their relative importance [52]. When the number of unexplained variation is low, it indicates that no fundamental variable is missing (number of variables was sufficient for explaining variation in data set) otherwise, it explains that nondeterministic fluctuations are in effect and more variables must be included in the analysis [48].

### Distribution modelling

In this study, we used GLM (Generalized linear model) which is basically a generalization of ordinary least squares regression [8] and GBM (Gradient Boosting Machine), a boosted decision trees method [58], as a more complicated algorithm which has good performance in SDM [59–61]. GLM and GBM were used to create potential distribution models of gray almond, using combination of different variable groups including climatic variables (C.P), climatic and edaphic variables (C.E.P), climatic and topographic variables (C.T.P) and finally climatic, edaphic and topographic variables (C.E.T.P). Weight of occurrence points were equivalent in all models [61]. Here, we used 10-fold cross-validation for each approach [7, 62]. Modelling process was performed using ‘biomod2’ package [63] in R platform [51].

Models were validated by calculating the area under the receiver operating curve (AUC) [64], true skill statistic (TSS) [65], sensitivity (true positive rate) and specificity (true negative rate) based on 10-fold cross-validation on the calibration data set [12, 66, 67]. AUC evaluates the ability of a model to distinguish between locations that a species is present, versus those that is not present. The performance score of the measured AUC ranged between 0–1, where 1 is a perfect score and the model is ideal, 0.5 represents a model of random selection and the results under 0.5 is reflects an unacceptable model [68]. TSS ranges between +1 (the best result) to -1 (the worst result) and calculates by (sensitivity + specificity) -1 [65]. Particularly, ROC plot has received considerable attention in SDM studies because it considered both sensitivity and specificity for all available thresholds [65] and provides a single measure of overall accuracy that is not dependent upon a particular threshold [64, 67].

We used representative concentration pathway (RCP) 2.6 of “The Community Climate System Model” (CCSM4) of future climate scenario for year 2070 (average for 2061–2080) provided by National Center for Atmospheric Research of USA widely used by researchers [69] and has strong agreement with climatic condition of Iran [70] for each SDMs.

Continuous probability of presence produced by models (GLM and GBM) transformed to binary presence/absence data using individual thresholds [71, 72]. Thresholds were calculated based on the ROC plot approach in "biomod2" library [63] in R [51] that is determined by the shortest distance of the curve to the top-left corner in the ROC plot [63].

## Results

### Principle analysis components

In this study, the first two PCs accounted for 41.06% of total variance of data set (PC1, 27.46% and PC2, 13.6%). The first principal components (PC1) captured more variance (27.46%) than expected by chance and is completely distinguished from the other dimensions. In PC1, solar radiation and isothermality explain the greatest proportion, and in PC2, precipitation of driest quarter and temperature seasonality explain the greatest proportion, respectively. The second to fifth components of environmental variables showed eigenvalues exceeding 1. The first six components explain 80.83% of the total variance, meaning lack of significant differentiation between components and mild differentiation between principal component 1 and others. In other words, overall combination of environmental variables affects the presence of gray almond.

**Fig 2:**
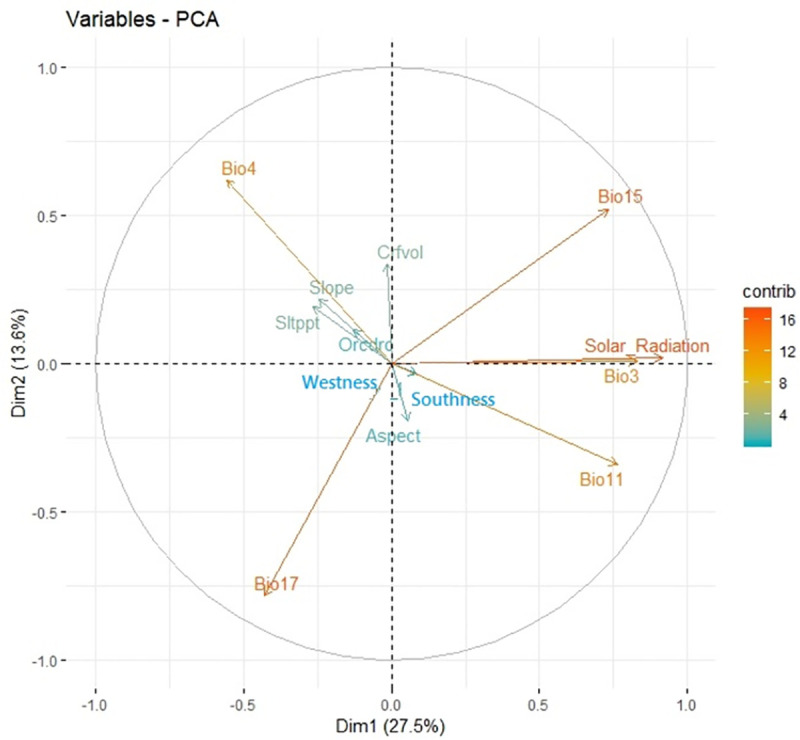
Visualizations of environmental conditions of *P*. *eburnea* occurrence locations in Iran by PCA analysis, which summarize variation among the environmental variables.

In the graph of variables (Fig 2), positive correlated variables point to the same sector and negative correlated variables point to opposite section of the graph. The results showed that solar radiation and isothermality (Bio3) are strongly correlated and overlap close to axis 1. Principal-components analysis (PCA) indicated that presence of *P*. *eburnea* in the study area is correlated with several important environmental variables that make a suitable multidimensional niche.

### Hierarchical partitioning

**Fig 3:**
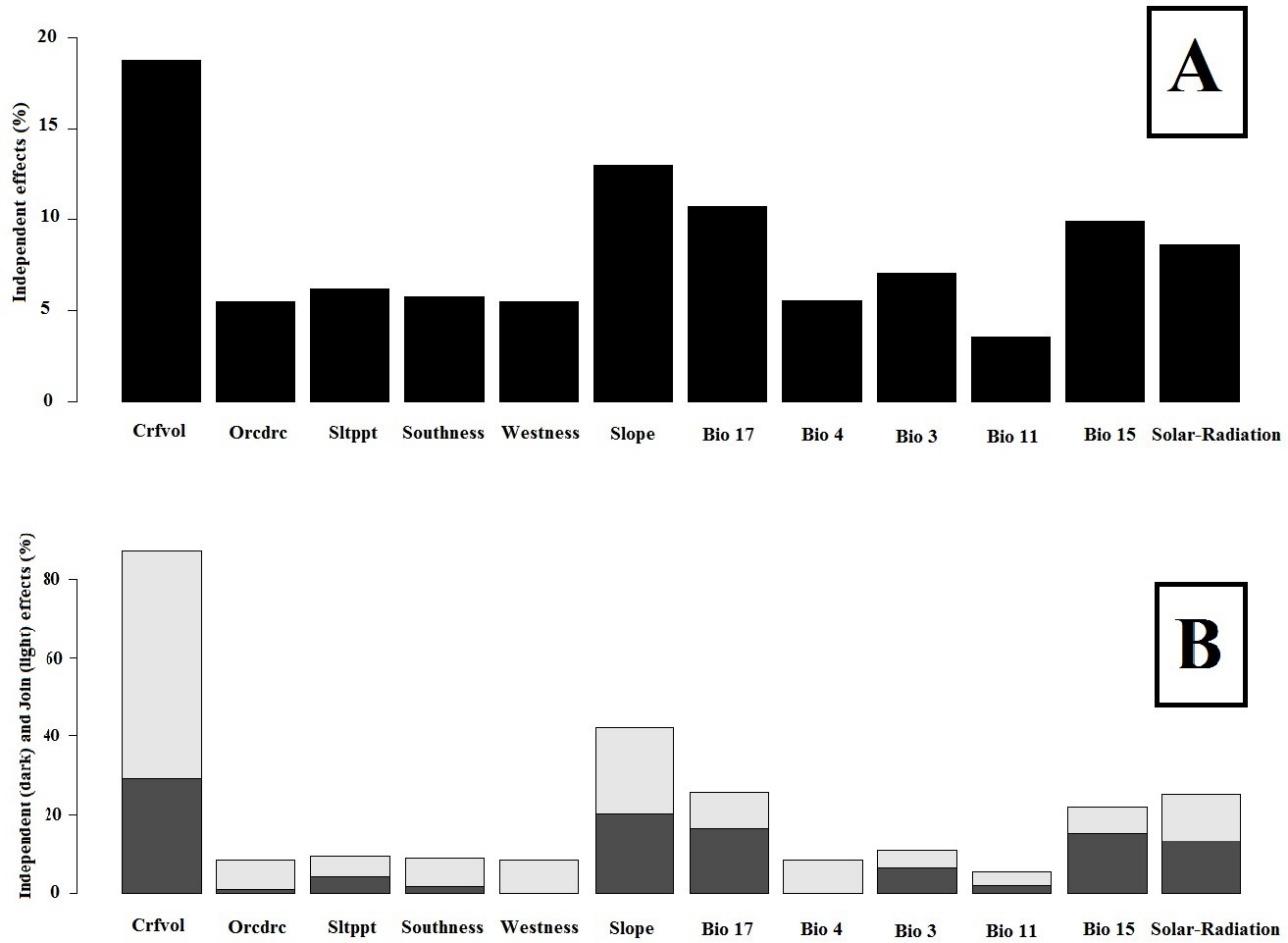
The independent (A), (given as the percentage of the total explained variance) and joint contributions (B), its independent contribution (dark color) and its conjoint contribution with all other variables (light color) of the variable variables for *P*. *eburnea*, as estimated from hierarchical partitioning.

The independent and joint contribution of each variable in presences and absences of gray almond was determined by HP (Fig 3). Results showed coarse fragments (volumetric) of soil and slope are the most important independent environmental variables, respectively and their joint effect is amplified.

In the hierarchical partitioning analyses, the independent effects of all included environmental variables were statistically significant. The highest independent contributions belong to coarse fragments (volumetric) of soil (crfvol), slope and precipitation of driest quarter (Bio17), respectively and the lowest contributions belong to mean temperature of coldest quarter (Bio11) and organic carbon content (orcdrc), respectively (Table 2).

**Table 2:** Results of the randomization tests for the independent contributions of separate variable variables in hierarchical partitioning for explaining variation of *P*. *eburnea* (Z.scores are computed as (observed—mean (randomizations))/sd(randomizations), and statistical significance (*) is based on upper 0.95 confidence limit (Z > = 1.65).

Environmental variables	Observed	Z.score
Crfvol	29.19	149.96 *
Orcdrc	8.54	41.50 *
Sltppt	9.58	35.29 *
Southness	8.98	64.15 *
Westness	8.56	44.81 *
Slope	20.20	50.54 *
Bio17	16.65	128.88 *
Bio4	8.59	60.31 *
Bio3	10.95	38.88 *
Bio11	5.50	21.36*
Bio15	15.38	99.16 *
Solar radiation	13.37	105.64 *

### Variation partitioning

For determining VP, different combinations of groups of variables were performed according to equations below: (X1, X2 and X3 represent the followings: X1 = group of edaphic variables, X2 = group of topographic variables and X3 = group of climate variables).

[a+d+f+g] = X1

[b+d+e+g] = X2

[c+e+f+g] = X3

[a+b+d+e+f+g] = X1+X2

[a+c+d+e+f+g] = X1+X3

[b+c+d+e+f+g] = X2+X3

[a+b+c+d+e+f+g] = All

**Fig 4:**
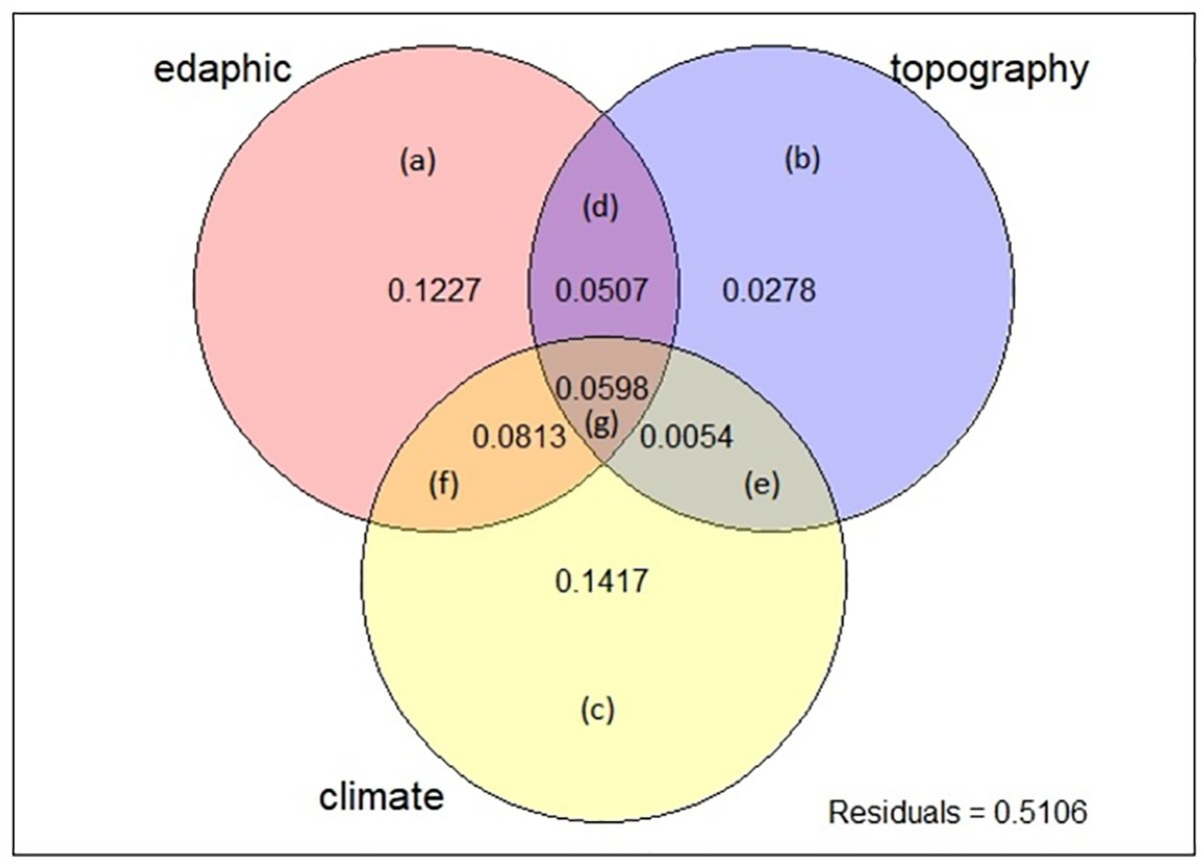
Results of variation partitioning of *P*. *eburnea* in terms of the fractions of variation is explained. In a venn diagram, each circle shows how much of the variations of response variables is explained by each group of variables and overlap areas show the joint contribution of different variables. It must be considered the size of circles and overlaps in figure did not scale to their numerical values (variation of the environmental data is explained by three groups of explanatory variables: X1 = group of edaphic variables, X2 = group of topographic variables and X3 = group of climate variables. Residuals are undetermined variation and a, b and c represent unique effects of edaphic, topography and climate variables, respectively; while d, e, f and g are fractions indicating their joint effects).

Results of partitioning of variation using these environmental variables in RDA showed that total variation (SS) sums up to 122.7 and Variance is 0.2494. In the case of the VP results, the total explained variation in *P*. *eburnea* environment data was obtained by regressing with the selected statistically significant variables (p<0.05) of the three groups of environmental variables (climatic, edaphic and topographic variables) and consequently, the residual amount calculated (Fig 4). (The size of circles and overlaps in Fig 4 did not scale to their numerical values).

Variation partitioning of the data set showed that 48.94% of total variation explained by used environmental variables. The portion of each group of environmental variables in this amount of justified variance is different. that climate, explained more variance (14.17%) in *P*. *eburnea* occurrence than topography (2.78%), soil (12.27%), climate and soil together (8.13%), climate and topography together (0.54%), soil and topography together (5.07%), soil, topography and climate together (5.98%). In general, climate variables by themselves were more powerful than the other variables and groups of variables together in explaining the variations. The results showed that 51.06 percentage of variation cannot be explained by environmental variables and it is related to biotic variables, the interactions between biotic and abiotic variables.

### Species distribution modelling

Results of assessing effects of different combinations of environmental variables to GLM and GBM showed that sensibility of different accuracy criteria to input variables are different. In GLM, area under the curve (AUC) scores ranged from 0.91–0.94 and specificity ranging 83.7–90.24. Also, sensitivity and true skill statistic (TSS) ranged from 83.9–92.84 and 0.67–0.75, respectively, which represented significant difference among, models accuracy. The lowest AUC score, TSS, sensitivity and specificity were observed when we used only climatic variables (Table 3).

**Table 3:** Comparison of area under the curve (AUC), true skill statistic (TSS), sensitivity and specificity statistics using 10 fold cross-validation for each GLM and GBM approaches using different fractions of environmental variables in modeling potential habitat suitability of gray almond in Iran (C.P: Climatic variables; C.E.P: Climatic and edaphic variables; C.T.P: Climatic and topographic variables; C.E.T.P: Climatic, edaphic and topographic variables).

Model	Accuracy criteria	C.P	C.E.P	C.T.P	C.E.T.P
GLM	AUC	0.91	0.93	0.94	0.94
	TSS	0.67	0.72	0.75	0.75
	Sensitivity	83.9	90.78	92.26	92.87
	Specificity	83.7	89.85	89.29	90.24
GBM	AUC	0.92	0.94	0.94	0.95
	TSS	0.85	0.86	0.88	0.89
	Sensitivity	86.13	91.3	93.69	93.87
	Specificity	87.29	89.63	91	93.7

In GBM, AUC score and TSS ranged from 0.92–0.95 and 0.86–0.89, respectively. Results indicated there are not tangible differences in AUC score and TSS using different groups of environmental variables. In all fractions, TSS and AUC score for GBM were greater than GLM. Sensitivity ranged from 86.13–93.87 that represented significant differences using different groups of environmental variables by GBM. Specificity ranged from 87.29–93.7 and it was the highest number, using only climatic variables (Table 3).

### Climate change impacts

Results of using different environmental predictors combinations in predicting potential distribution of gray almond using CCSM4, RCP 2.6 in 2070 by GLM and GBM showed that using different fractions of environmental variables has a strong impact on the estimated potential of future range size. Using climatic variables alone led to predicting a major reduction of the potential distribution of gray almond but by including topographic and edaphic variables besides climatic variables, we observed a minor reduction in predicting potential distribution of gray almond in future (Table 4).

**Table 4:** Current and predicted future potential distribution and changes in habitat suitability under climate change condition for gray almond using different fractions of environmental variables by GLM and GBM; units is km^2^ (C.P: Climatic variables; C.E.P: Climatic and edaphic variables; C.T.P: Climatic and topographic variables; C.E.T.P: Climatic, edaphic and topographic variables).

Model	Group of variables	Loss (%)	Gain (%)	Range Change (%)	Current Range Size	Future Range change
GLM	C.P	84.23	3.45	-80.78	763349	616633
	C.E.P	62.86	8.6	-54.26	857789	465436
	C.T.P	63.15	6.7	-56.45	844635	476796
	C.E.T.P	46.01	9.52	-36.49	869066	317122
GBM	C.P	81.45	4.12	-77.33	740177	572378
	C.E.P	35.12	5.85	-29.27	831042	243245
	C.T.P	29.67	5.3	-24.37	839083	204484
	C.E.T.P	23.28	7.11	-16.17	852893	137912

**Fig 5:**
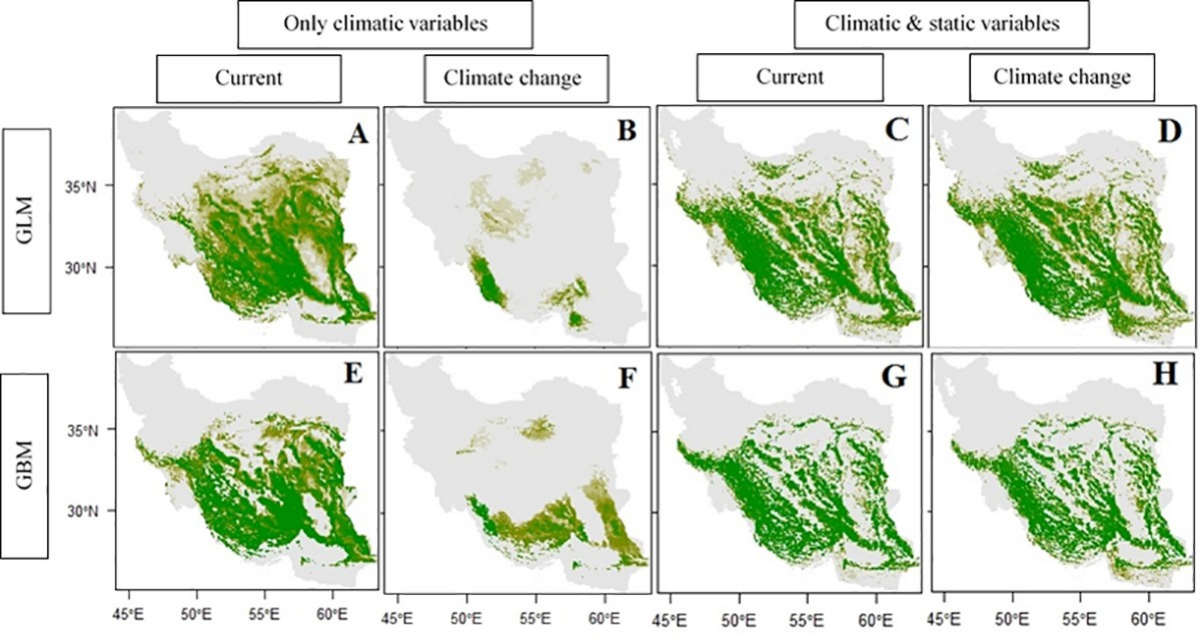
Projections using a purely dynamic model (based on climate only) versus static + climatic model by GLM and GBM. (A: Current distribution using climatic variables by GLM; B: Future distribution using climatic variables by GLM; C: Current distribution using climatic and static variables by GLM; D: Future distribution using climatic and static variables by GLM; E: Current distribution using climatic variables by GBM; F: Future distribution using climatic variables by GBM; G: Current distribution using climatic and static variables by GBM; H: Future distribution using climatic and static variables by GBM).

Projected effects of climate change using RCP 2.6 in 2070 on prediction distribution change of gray almond (Fig 5) shows big differences in the results between different groups. Results highlight in the margin of potential distribution especially in lower latitude, there would be more possibility for distribution by GLM and GBM.

## Discussion

Our results indicated that variable selection has strong influence on predicting species distribution and model accuracy especially in case of climate change, which is commonly, used for conservation and management purposes. Here, species distribution models calibrated with climatic, topographic and edaphic variables (C.T.E.P) performed better than the others (C.P, C.E.P and C.T.P) and models were more precise (Table 3) and more realistic (Fig 5). The lowest accuracy of GLM and GBM (Table 3) was achieved using only climatic variables, which are commonly used in isolation to infer future species distributions. Since climate is not the only factor affecting distribution of species, it cannot adequately represent habitat suitability completely without considering other effective environmental variables in correlative models [22, 73]. Using only climatic variables in species modelling which their distribution is limited by non-climatic variables leads to increasing risk of overfitting, under or over estimation of changes in potential distribution [72] and probability of extinction under climate change conditions [74, 75].

Here, our results showed static variables increased accuracy and reliability of modelling current habitat suitability (Table 3) and predicting habitat suitability of gray almond under climate change conditions (Table 4). Because, using appropriated static variables beside dynamic variables lead to more explaining niche species [61] and increasing accuracy of predictions in modelling [20, 22, 24]. Static variables play an important role in determining distribution of species. For increasing accuracy and reliability of models, it is better to include static variables, such as land cover and land use in SDM than to exclude them [31] as showed on investigations on butterflies [13].

In this study, we used principal component analysis (PCA), variation partitioning (VP) and hierarchical partitioning (HP) methods to identify relevant species–environment relationships for *P*. *eburnea*). Recognition of the proportion of each variable and group of variables in constituting a suitable habitat is helpful for better tracing and evaluating the changes in habitats. Climate information was the most important environmental group of variables for settling *P*. *eburnea* in different habitats (Figs 3 and 4). Climate characteristics are influencing factors in delimiting distribution of plants. Minimum temperatures not only have controlling roles in physiologic functions of plants [76, 77], but also affect the susceptibility of species to attack by pathogens and pests [78] and their competing and coexisting ability in interspecies interactions [79, 80]. Joint effects of environmental variables induce additive impacts on some aspects of environment. For example, combination of the effects of the solar radiation and seasonal temperature differences during winter and summer lead to different environmental conditions [81], which is especially relevant for plants which start their germination and flowering in late winter, such as species of *Prunus* and other rosaceae species. Results of this research indicated cumulative effects of soil depth (Crfvol) and slope on *P*. *eburnea* presence in specific habitats (Fig 3). Cumulative effects can be synergistic (amplifying) or antagonistic (decreasing) in their influence. Therefore, interactions amongst different groups of variables are important and must be taken into account when suitable habitat for species is determined. For example, temperature and precipitation are two main factors of climate that are both influenced by elevation directly. Solar radiation is dependent on latitude and affects photoperiod of plants, which controls most of physiological functions and biomass producing [82]. The high degree of overlap between climate and soil (8.13%) is at least partly because of the dependence of soil (formation, recycling, weathering and erosion) on climate [83]. In addition, results showed that soil organic carbon explained high portion of total variations in distribution of *P*. *eburnea* (Figs 3 and 4). Importance of soil organic carbon content (Figs 2 and 3) derived from its role in the determining accessibility of water and nutritious elements [84] and consequently to wider occurrence of plant species [85]. Similar effects result from soil texture, which facilitates nitrogen and phosphorous uptake [76, 86]. Soil properties by affecting on water and nutrients availability [87], physical support for root growth and establishment in habitats in most of plants [88, 89] play a key role in determining suitable habitats which improve accuracy of predictions [13, 90] indicated including edaphic variables, along with climatic variables led to more realistic predictions of the current distribution of shrub species. Actually, adding relevant edaphic variables to modelling by increasing probability of recognizing suitable habitats, resulted to improvement of model performance and led to more accurate mapping [90, 91]. Topographic variables by effecting on other environmental factors such as soil properties [73] play an important role in limiting species distribution [72]. Using efficient-scale topography variables in prediction of future distribution of species would help to recognize refugia (shelter) and therefore to increase the accuracy of the species range change prediction [73].

Results of this study showed that abiotic factors contributed to robustly explain the occurrence of *P*. *eburnea* in its native habitats and the remainder is related to biotic variables and interactions between biotic and abiotic variables that were not accounted for in our analyses. Our results indicated that regarding static variables in SDM especially in predicting impacts of climate change on species occurrences changed the predictions significantly (Fig 5). Topographic characteristics [73] and soil properties at a large scale affect plant growing conditions are key factors in predicting current and improving prediction of future potential distribution; therefore, considering only climatic variables (Table 4) (Fig 5) and ignoring topographic and edaphic heterogeneity lead to over prediction of shifts and extinction rate of species under climate change conditions [73].

When inferring and interpreting of SDM, one needs to consider that limiting factors of species distribution are far more than environmental factors and colonization and extinction dynamics may be relevant to species distributions, even though, these variables may not address those relations. It is almost impossible to measure the true niche of a species completely, because, we cannot measure and quantify all factors (biotic, abiotic and complex interactions between them) which influence presence (and absence) and distribution range of species and only we usually can get an overview on much of the conditions that make up niche. Therefore, extrapolating results from correlative modelling approaches should always be made with caution and choosing modelling variables must be prioritized [92].

## Conclusion

The results of this study indicate that use of solely climatic variables would exaggerate the prediction effects of climate change on the potential distribution of species range and that non-climatic variables, such as soil and topographic characteristics are important factors that can also constrain the rate of climate-induced range expansion. This study showed the importance of including the appropriated combination of variables for habitat suitability modelling and prediction effects of climate change on it. Our results indicated using static environmental variables in addition to climatic variables to modeling, increased overall accuracy criteria of models and the results were more realistic. Although, some studies indicated that using only climatic data may provide an effective and efficient approach for primary evaluations of habitat suitability [28], our results indicated that using only climatic variables overestimate the impacts of climate change on species distributions. Disentangling of broad climatic drivers from microhabitat and soil factors in determining species distributions is of paramount importance, because, it will provide information on potential impacts of future climate change and the possible mechanisms leading to altered plant diversity (alpha, beta and gamma diversity).

## Supporting information

S1 File (RAR)
